# Effectiveness of low-level laser on carpal tunnel syndrome: A meta-analysis of previously reported randomized trials

**DOI:** 10.1097/MD.0000000000004424

**Published:** 2016-08-07

**Authors:** Zhi-Jun Li, Yao Wang, Hua-Feng Zhang, Xin-Long Ma, Peng Tian, Yuting Huang

**Affiliations:** a Department of Orthopedics, Tianjin Medical University General Hospital; b Department of Oncological Surgery, Tianjin Nankai Hospital, Tianjin Integrated Traditional Chinese and Western Medicine Hosptial; c Department of Orthopedics, Tianjin Hospital, Tianjin, People's Republic of China; d Cancer & Immunology Research, Children's Research Institute, Children's National Medical Center, Washington DC.

**Keywords:** carpal tunnel syndrome, low-level laser, meta-analysis

## Abstract

**Background:**

Low-level laser therapy (LLLT) has been applied in the treatment of carpal tunnel syndrome (CTS) for an extended period of time without definitive consensus on its effectiveness. This meta-analysis was conducted to evaluate the effectiveness of low-level laser in the treatment of mild to moderate CTS using a Cochrane systematic review.

**Methods:**

We conducted electronic searches of PubMed (1966–2015.10), Medline (1966–2015.10), Embase (1980–2015.10), and ScienceDirect (1985–2015.10), using the terms “carpal tunnel syndrome” and “laser” according to the Cochrane Collaboration guidelines. Relevant journals or conference proceedings were searched manually to identify studies that might have been missed in the database search. Only randomized clinical trials were included, and the quality assessments were performed according to the Cochrane systematic review method. The data extraction and analyses from the included studies were conducted independently by 2 reviewers. The results were expressed as the mean difference (MD) with 95% confidence intervals (CI) for the continuous outcomes.

**Results:**

Seven randomized clinical trials met the inclusion criteria; there were 270 wrists in the laser group and 261 wrists in the control group. High heterogeneity existed when the analysis was conducted. Hand grip (at 12 weeks) was stronger in the LLLT group than in the control group (MD = 2.04; 95% CI: 0.08–3.99; *P* = 0.04; *I*^2^ = 62%), and there was better improvement in the visual analog scale (VAS) (at 12 weeks) in the LLLT group (MD = 0.97; 95% CI: 0.84–1.11; *P* < 0.01; *I*^2^ = 0%). The sensory nerve action potential (SNAP) (at 12 weeks) was better in the LLLT group (MD = 1.08; 95% CI: 0.44–1.73; *P* = 0.001; *I*^2^ = 0%). However, 1 included study was weighted at >95% in the calculation of these 3 parameters. There were no statistically significant differences in the other parameters between the 2 groups.

**Conclusion:**

This study revealed that low-level laser improve hand grip, VAS, and SNAP after 3 months of follow-up for mild to moderate CTS. More high-quality studies using the same laser intervention protocol are needed to confirm the effects of low-level laser in the treatment of CTS.

## 1 Introduction

Carpal tunnel syndrome (CTS) is an important mononeuropathy that is mainly caused by entrapment of the median nerve by a swollen transverse carpal ligament resulting from chronic inflammation. The change in the median nerve in CTS is a process. Early compression causes a block in venous outflow leading to the nerve becoming hyperemic and edematous;^[1]^ this process is followed by inflammatory reaction, fibrosis, demyelination, and axonal loss over the next 30 days.^[2]^ Additionally, increased expression of prostaglandin E2, vascular endothelial growth factor,^[3]^ and interleukin-6^[4]^ might play a role in CTS. Diagnosis is based on clinical symptoms, physiological tests, and electrodiagnostic examination.^[5,6]^ Clinical symptoms and signs are characterized by numbness and tingling of the first 3 fingers and the radial side of the ring finger, nocturnal awakening from pain, and weakness or atrophy of the thenar muscle. Phalen's maneuver and Tinel's sign are positive in some patients. Nerve conduction studies show longer latency and slower conduction velocity than in normal conditions.

For serious cases, surgical intervention is an effective choice for relieving pressure around the median nerve, although there is a risk of recurrence.^[7,8]^ Recurrent symptoms of CTS have been shown to occur in 0% to 19% of patients following surgery, and up to 12% of cases require re-exploration.^[9]^ The natural history of CTS typically progresses slowly, and some patients can recover spontaneously.^[10]^ Therefore, conservative treatments are welcome in mild and moderate patients and have less expense and less frequent complications. Nonsurgical treatments are available, including exercise, wrist splinting, nonsteroidal anti-inflammatory drugs, local injection of corticosteroid, and ultrasound (US).

Low-level lasers were first studied by Padua et al,^[11]^ and studies have shown that increasing myelin production and reducing retrograde degeneration of motor neurons were found in a rat spinal cord crushing model.^[12]^ Other possible mechanisms of the benefits of low-level lasers include anti-inflammatory effects,^[13]^ selective inhibition of nociceptive activation at peripheral nerves,^[14]^ increased ATP production and cellular respiration,^[15,16]^ and improvement of blood circulation to remove algesic substances.^[13,17]^ Weintraub suggested that 9 J of energy over 5 points (7–15 treatments) reversed CTS in 77% of cases.^[18]^ However, these studies were uncontrolled. The safety profile of LLLT was later established for clinical use.^[19]^

In recent years, some placebo-controlled studies have shown beneficial effects of LLLT on clinical and electrophysiological parameters in the treatment of CTS.^[20–25]^ However, these findings are not consistent because of different laser intervention protocols. Moreover, the functional mechanism of low-level lasers is not clear, and some studies suggested that laser irradiation did not change the functional properties of peripheral nerves.^[26,27]^ Thus, this study was conducted to critically review and summarize the literature regarding low-level lasers to obtain a clear answer concerning the effectiveness of LLLT as a promising treatment for CTS.

## 2 Methods

### 2.1 Search strategy

Electronic searches of PubMed (1966–2015.10), Medline (1966–2015.10), Embase (1980–2015.10), and ScienceDirect (1985–2015.10) were performed to identify trials according to the Cochrane Collaboration guidelines. We used the following search terms and different combinations of the terms: “low level or low intensity,” “laser,” “carpal tunnel syndrome” with the Boolean operators AND or OR. Manual searches including those of the reference lists of all the included studies were used to identify trials that the electronic search might have failed to identify. There was no restriction on language. Two reviewers independently assessed the titles and abstracts of all the reports identified by the electronic and manual searches. When inclusion was unclear based on the abstracts, full text articles were retrieved. Disagreements were resolved through discussion. This study is a meta-analysis, which need not the ethics committee or institutional review board to approve the study.

### 2.2 Inclusion and exclusion criteria

Trials with the following characteristics were included: (1) randomized clinical trials; (2) comparison of low-level laser with or without splinting for CTS; (3) mild or moderate CTS; (4) full text articles; and (5) available data to be used. Exclusion criteria were as follows: (1) patients who received nonsteroidal anti-inflammatory drugs, and oral corticosteroids or local injection of corticosteroids before LLLT; (2) studies comparing LLLT with other conservative treatment; (3) articles that were duplicate reports of earlier trials, post-hoc analyses of randomized controlled trials (RCTs) data, and articles for which we were unable to obtain the full text.

### 2.3 Quality assessment

A quality assessment was conducted according to the Cochrane Collaboration's tool for assessing the risk of bias and included the following key domains: adequate sequence generation, allocation of concealment, blinding, incomplete outcome data, and an absence of selective reporting and other bias. Disagreements were resolved by discussion or by consultation with the senior reviewer.

### 2.4 Data extraction

Two authors independently extracted the data from the included articles. Data regarding the authors, year, patient demographics, inclusion and exclusion criteria, interventions, outcomes, and follow-up tests for each group were extracted. We attempted to contact the authors for supplementary information when the reported data were inadequate.

### 2.5 Data analysis and statistical methods

The meta-analysis was undertaken using RevMan 5.1 for Windows (Cochrane Collaboration, Oxford, United Kingdom). Statistical heterogeneity was assessed using a standard chi-square test (the statistical heterogeneity was considered significant at *P* < 0.05) and the *I*^2^ statistic (*I*^2^ value of 50% or higher was considered to indicate substantial heterogeneity).^[28]^ When heterogeneity occurred, the pooled data were meta-analyzed using a random-effects model. Otherwise, a fixed-effects model was used for the analysis. The mean difference (MD) and 95% confidence interval (CI) were calculated for the continuous outcomes.

## 3 Results

### 3.1 Literature search

**Figure 1:**
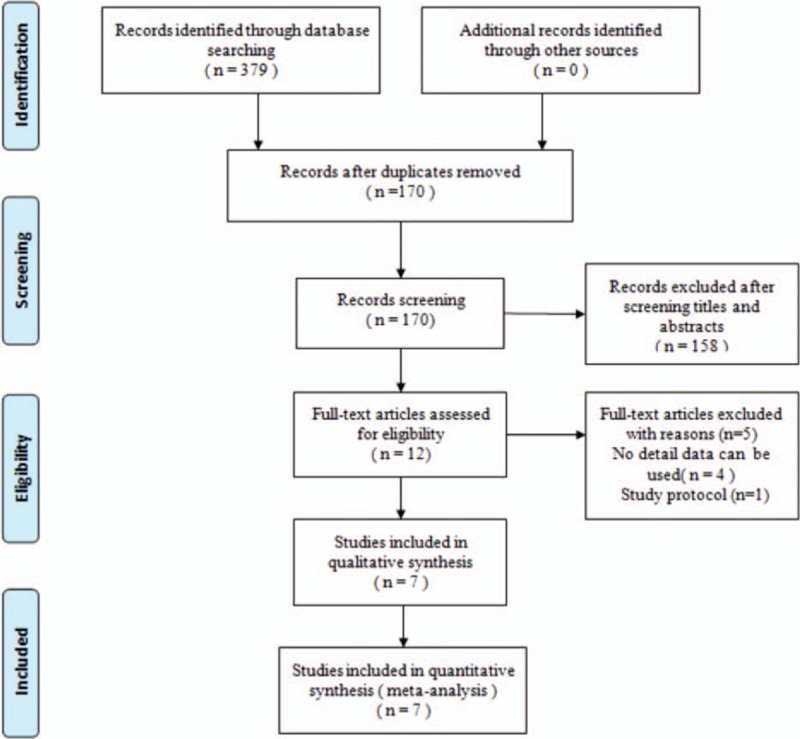
Flowchart showing the identification and selection of studies.

Figure 1 shows the flowchart of the study selection and inclusion process. A total of 170 potential studies were identified with the first search strategy. Of these, 161 reports were excluded, based on the eligibility criteria. One RCT by Lazovic was excluded because no available data can be pooled to calculate together.^[29]^ No additional studies were obtained after the reference review. The search strategy ultimately identified 7 randomized clinical trials satisfying the predefined inclusion criteria; there were 270 wrists in the laser group and 261 wrists in the control group.^[22–24,30–33]^ Individual patient data were available from these articles, except for data for the subjects lost to follow-up.

### 3.2 Study characteristics

The characteristics of the included studies are summarized in Table 1. Statistically similar baseline characteristics were observed between the 2 groups. The sample sizes in the studies ranged from 15 to 141 wrists. Among these studies, a splint was used in the patients in 3 studies.^[22,30,33]^ The laser treatment methods were different in all of these studies.

**Table 1:**
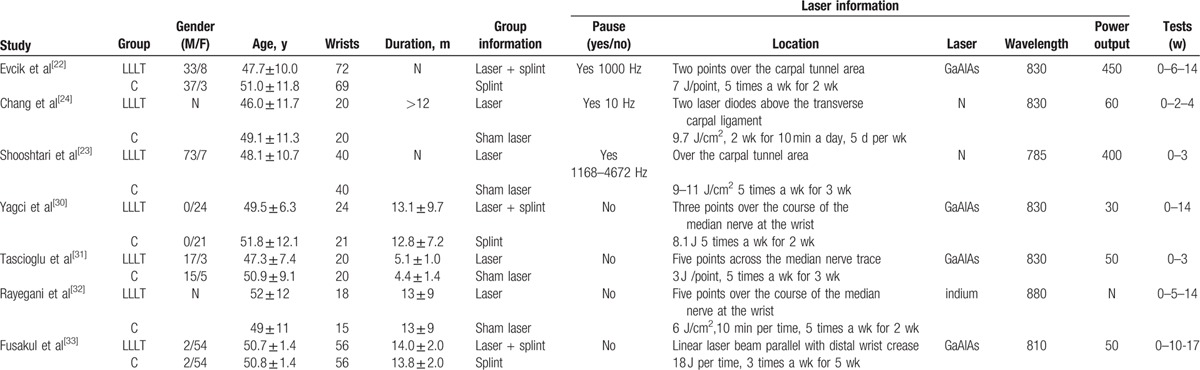
Characteristics of included studies.

### 3.3 Risk of bias assessment

**Figure 2:**
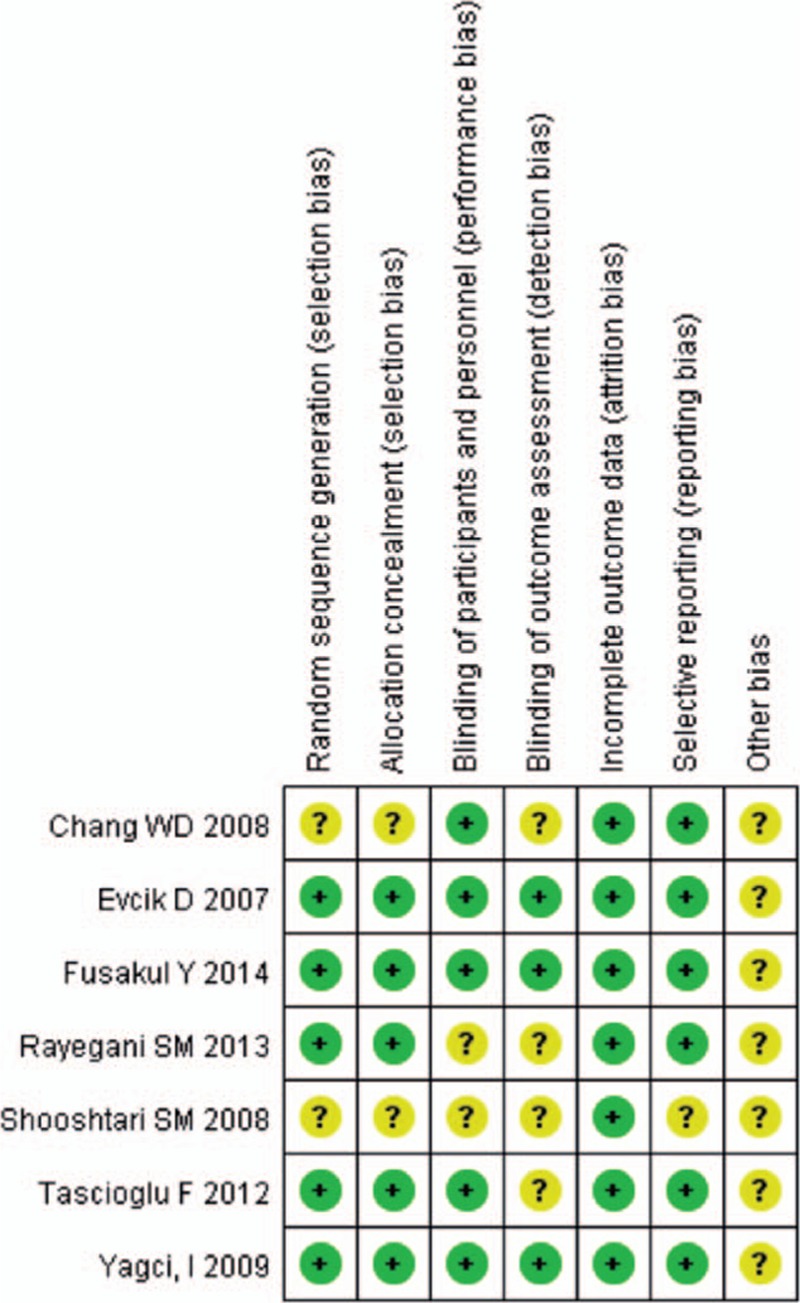
Methodological qualities of the included studies.

Random sequence generation and allocation were not described in 2 studies.^[23,24]^ The blindness of the participants and personnel was not clear in 2 studies,^[23,32]^ and the blindness of the outcome assessment was not described in 4 studies.^[23,24,31,32]^ The details of the methodological quality of the included studies are presented in Fig. 2.

### 3.4 Outcomes for meta-analysis

The clinical parameters of hand grip strength, visual analog scale (VAS), symptom severity scores (SSS), and functional status scores (FSS) of the patients were calculated according to the test time. Because different follow-up times for clinical or electrophysiological tests were adopted in the included studies, we defined a “short” time as less than 6 weeks after treatment and a “long” time as 12 weeks. The meta-analysis results of clinical parameters are summarized in Table 2. No significant differences between the 2 groups were observed in most of the parameters with the exception of hand grip (long) and VAS. The hand grip (long) was stronger in the LLLT group than in the control group (MD = 2.04; 95% CI: 0.08–3.99; *P* = 0.04; *I*^2^ = 62%);^[22,30,33]^ better improvements in VAS (long) were found for the LLLT group (MD = 0.97; 95% CI: 0.84–1.11; *P* < 0.01; *I*^2^ = 0%).^[32,33]^ However, the study by Fusakul et al^[33]^ was weighted as >95% in the calculation of hand grip strength and VAS at 12 weeks.

**Table 2:**
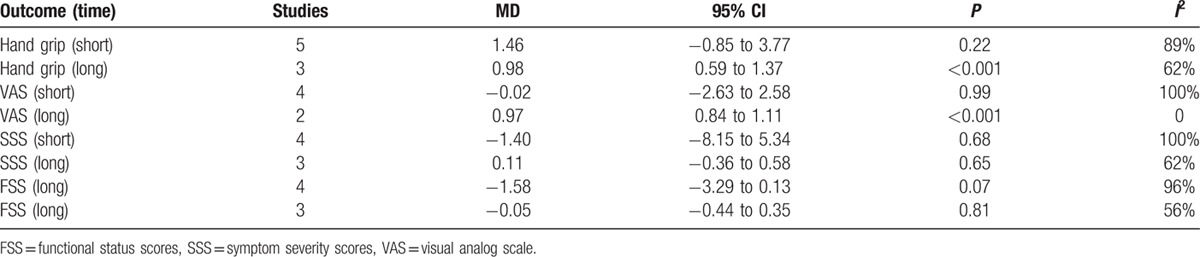
Results of clinical parameters.

Different electrodiagnostic parameters examining the effects of lasers on nerves were tested and are summarized in Table 3. Similar to the clinical tests, most of the nerve conduction studies showed no significant differences between the 2 groups with apparent heterogeneity. The only significant difference was noticed for SNAP, and the study by Fusakul et al occupied >95% of the weight. The SNAP (long) was better in the LLLT group than in the control group (MD = 1.08; 95% CI: 0.44 to 1.73; *P* = 0.001; *I*^2^ = 0%).^[30,32,33]^

**Table 3:**
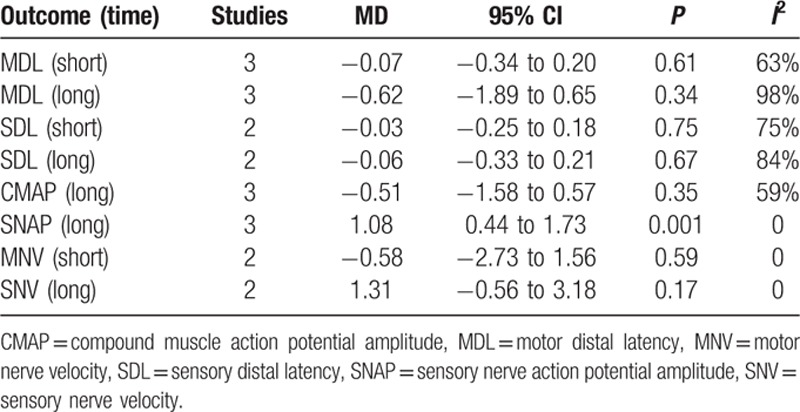
Results of electrodiagnostic testing.

## 4 Discussion

Although LLLT has been reportedly used in clinical practice with good performance, no statistically significant differences were found in most clinical parameters or nerve conduction studies between the groups in our meta-analysis based on 7 randomized controlled trials. This study revealed that low-level laser improves hand grip, VAS, and SNAP after 3 months of follow-up for mild to moderate CTS. No statistically significant differences were found in other clinical parameters or nerve conduction studies between these 2 groups.

Whether significant differences were found in these parameters, an important problem is that high heterogeneity existed in most of the calculations, which would lower the persuasive power of this meta-analysis. Many factors would influence the precision of the results, such as heterogeneous participants, different interventions, and different follow-up times for conducting clinical or electrophysiological tests. In the included studies, the inclusion criteria of the patients were similar; mild to moderate cases were recruited without surgery of the wrist, rheumatoid arthritis, a history of metabolic disease, paralyzed limbs, or similar conditions.

The LLLT factors were important, including wavelength, power, frequency, pulse or not, action position, and treatment schedule.^[34,35]^ Different laser irradiation doses for patients in the included studies were adopted; the doses are expressed as energy from 2.7 to 11 J for each point or as total energy from 81 to 300 J for the entire treatment. Three or 5 points over the course of the median nerve at the wrist was the most commonly used action position, whereas 2 laser diodes above the transverse carpal ligament were used in the study of Chang et al.^[24]^ These differences resulted in heterogeneity in the meta-analysis. We were unable to find a good method to conduct subgroup analyses based on 1 factor. The difficulties in the analysis made it impossible to determine which low-level laser treatment protocol was best and should be adopted.

Another factor that contributed to high heterogeneity is the test time during the follow-up. The evaluation times were different in the included studies. In our study, long follow-up tests were performed 3 months after treatment and short tests were conducted immediately, 2, 4, or 5 weeks after treatment. Calculating the data from different test times together results in heterogeneity. Subgroup analyses based on different test times are a good choice to allow further understanding, although these analyses could not be performed in this study because of the number of inadequate studies. Detecting the actual effects of low-level laser treatment on CTS during different processes is difficult.

In addition to the limitations mentioned above, the application of a splint in some studies would influence the results. Immobilization of the wrist in a neutral position with a splint could maximize carpal tunnel volume, facilitating the release of pressure on the median nerve.^[36]^ The effect of a splint on CTS might confuse the power of LLLT. Additional RCTs with a similar laser treatment protocol are needed to minimize bias and confirm the effect of LLLT in the treatment of CTS.

## 5 Conclusions

The results of this review show that low-level laser improves hand grip, VAS, and SNAP after 3 months of follow-up for mild to moderate CTS. However, more high-quality studies with the same laser intervention protocol and follow-up time are needed to decrease heterogeneity and to confirm the effects of LLLT on CTS. Besides, we also need double-blind studies to evaluate the effects of applying LLLT comparing with conventional therapies including anti-inflammatory medication on improving clinical and electrophysiologic findings in patients with mild to moderate CTS.
